# Study of Human Antimicrobial Peptides Active Against Some Bacteroidota Species of the Oral Cavity

**DOI:** 10.3390/antibiotics15010080

**Published:** 2026-01-13

**Authors:** Giusy Castagliuolo, Eugenio Notomista, Alessia Sordillo, Laura Barone, Dario Antonini, Francesco Renzi, Anna Zanfardino, Mario Varcamonti

**Affiliations:** 1 Department of Biology, University of Naples Federico II, 80126 Naples, Italy; giusy.castagliuolo@unina.it (G.C.); notomist@unina.it (E.N.); alessiasordillo2015@gmail.com (A.S.); barone-laura@libero.it (L.B.); dario.antonini@unina.it (D.A.); varcamon@unina.it (M.V.); 2 Research Unit in Biology of Microorganisms (URBM), Namur Research Institute for Life Sciences (Narilis), University of Namur, 5000 Namur, Belgium; francesco.renzi@unamur.be; 3 Le Fonds de la Recherche Scientifique-FNRS, Rue d’Egmont 5, 1000 Bruxelles, Belgium

**Keywords:** antimicrobial peptides (AMPs), bacteroidota, oral microbiome bacteria

## Abstract

The increasing problem of antibiotic resistance is a critical global health issue, necessitating the development of alternative therapeutic strategies to manage infections effectively. Among the promising solutions are human antimicrobial peptides (AMPs), naturally occurring molecules known for their broad spectrum of antimicrobial activity. **Background/Objectives:** This study investigates the potential of some AMPs, selected through a bioinformatic approach, as alternatives to conventional antibiotics, particularly focusing on their efficacy against species within the Bacteroidota phylum. These species, including pathogens such as *Porphyromonas gingivalis*, *Capnocytophaga ochracea*, and *Capnocytophaga canimorsus*, are well known for their roles in various human infections and related diseases. Non-pathogenic environmental species, such as *Flavobacterium johnsoniae*, are also included in this group, frequently used as a model organism. **Methods:** By analyzing the antimicrobial efficacy, mechanisms of action, and potential therapeutic applications of human AMPs, this research underscores their significance in addressing the challenge of antibiotic resistance. **Results:** This study identified three peptides, KTL24, LIR23, and MFP22, as particularly interesting. These peptides are derived from specific human proteins, namely SPI1, NAPSA and SCUB1. **Conclusions:** Their notable antimicrobial potential suggests that AMPs could serve either as a complementary treatment alongside traditional antibiotics or as a standalone therapy, mitigating the ongoing spread of antibiotic resistance and offering an alternative in global health strategies.

## 1. Introduction

Antimicrobial peptides (AMPs) are small, naturally occurring peptides that play a key role in the innate immune defense of organisms across all domains of life, including humans, plants, and microorganisms. They typically consist of 10–50 amino acids and exhibit broad-spectrum antimicrobial activity against bacteria, viruses, fungi, and parasites. Thanks to their amphipathic structure, positive net charge (+2 to +11), and high content of lysine and arginine, AMPs preferentially interact with negatively charged microbial membranes while generally sparing zwitterionic mammalian cells [1,2]. Once bound, AMPs can disrupt membranes via pore formation or translocate intracellularly to inhibit essential microbial processes. These properties, combined with their low propensity for resistance development, have made AMPs promising alternatives to conventional antibiotics in the fight against growing antimicrobial resistance, a problem that is particularly relevant in the oral cavity, a complex environment that hosts one of the densest and most diverse microbiotas in the human body [3].

It is estimated that over 700 bacterial species are present in the oral cavity, with a composition dominated primarily by the Firmicutes and Bacteroidota phyla, which together can represent up to 70–80% of the total microbial community [4]. While many of these species play a fundamental role in maintaining local homeostasis, some can acquire opportunistic traits and contribute to the development of diseases such as gingivitis, periodontitis, and caries [5].

AMPs, therefore, represent a potential therapeutic alternative capable not only of combating infection but also of modulating the microbial community, promoting the restoration of conditions and compositions closer to a healthy state.

The identification of novel oral microbiota-specific AMPs has recently been facilitated by the use of advanced bioinformatics strategies, such as in silico screening, structural predictions, and machine learning models, which allow for the rapid and efficient selection of promising peptides. This approach allows for the identification of molecules with targeted activity against clinically relevant oral bacteria, while minimizing side effects on the overall microbial ecosystem [6,7].

In this study, we focused on four human AMPs (KTL24, LIR23, PQR19, and MFP22) selected through an informatics screening, evaluating their antimicrobial activity against a targeted panel of Gram (−) species belonging to the phylum Bacteroidota, including both commensal and pathogenic strains.

*Porphyromonas gingivalis* and *Capnocytophaga ochracea* are well-established contributors to periodontal infections and have also been implicated in systemic conditions such as cardiovascular diseases and neurological disorders, while *Capnocytophaga canimorsus* infections have often been related to severe human systemic infections upon transmission from dogs and cats [8,9]. *P. gingivalis* is recognized as a keystone pathogen in chronic periodontitis and has been linked to systemic diseases, including Alzheimer’s disease and rheumatoid arthritis [10,11]. *C. ochracea* is an oral commensal that contributes to biofilm formation and produces immunosuppressive polysaccharides, and although less virulent, it has been implicated in extraoral infections in immunocompromised individuals [12,13,14]. *C. canimorsus*, typically transmitted by dog or cat bites, can cause severe systemic infections, including sepsis, meningitis, and endocarditis, mainly in immunocompromised hosts [15,16,17]. Its ability to evade host detection and cause stealth bacteremia makes it an excellent model for evaluating peptide efficacy.

We also included *Flavobacterium johnsoniae*, a non-pathogenic environmental model organism valued for its gliding motility, biofilm formation, and role in nutrient cycling within the Bacteroidota phylum and belonging to the Flavobacteriaceae family, the same as *Capnocytophaga* [18].

Finally, to broaden the panel, we evaluated the peptides against two biofilm-forming Gram (+) species belonging to the phylum Firmicutes: *Streptococcus mutans* and *Streptococcus oralis*. *S. mutans* is notorious for its cariogenic potential, fermenting dietary sugars into lactic acid that drives enamel demineralization [19]. *S. oralis*, while less cariogenic, plays a critical role as an early colonizer, facilitating adhesion through interactions with salivary glycoproteins and platelet receptors [20].

By combining computational pre-selection with experimental testing, this study aims to precisely characterize the antimicrobial and antibiofilm effects of selected human AMPs against clinically relevant Gram (−) and Gram (+) oral species, contributing to the development of novel anti-infective therapeutics.

## 2. Results

### 2.1. Identification of the Cryptic Antimicrobial Region in SPI1, NAPSA, SCUB1

**Figure 1:**
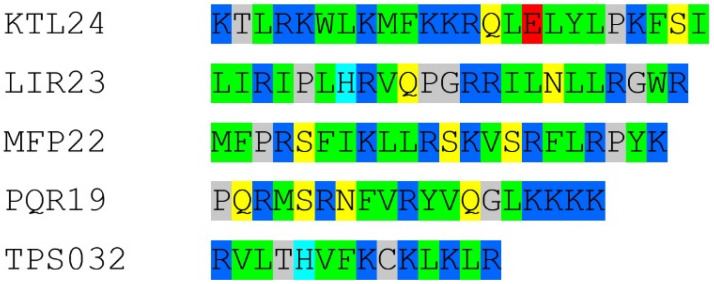
Sequences of the human cryptic AMPs colored by residue properties. Red, acidic; blue, basic; yellow, polar uncharged; green, hydrophobic; gray, borderline. The sequence of TPS032 is shown for comparison.

The four selected human cryptic AMPs share the typical composition of cationic AMPs, being rich in hydrophobic and cationic residues, as shown in Figure 1. They were previously identified by using an in silico method that assigns to a given peptide a score (A.S.) roughly proportional to the antimicrobial potency of the peptide [defined as Log (1000/MIC)] [21]. By plotting the A.S. values of all the possible peptides of the desired lengths inside a protein sequence of interest (sliding window analysis), it is possible to obtain a detailed map of the AMP-like regions inside the protein, as shown in Figure S1 for the peptides KTL24, LIR23, and MFP22. KTL24 is an internal region (Figure S1A) of the plasma serine protease inhibitor (UniProt: P05154), a secreted protein present in many body fluids and secretions. LIR23 (Figure S1B) is the first half of the activation peptide of Napsin A (UniProt: O96009), an extracellular protease likely involved in the maturation of pneumocyte surfactant proteins in the lung. The activation peptide is cleaved upon the conversion of the preprotein to the active protease. Interestingly, apart from the role in the secretion and regulation of Napsin A, to the best of our knowledge, no other function has been assigned to the activation peptide so far. MFP22 (Figure S1C) is the C-terminus of SCUB1, Signal peptide, CUB and EGF-like domain-containing protein 1 (UniProt: Q8IWY4), a protein highly secreted by platelets. PQR19 derives from a long (more than 45 residues) high-scoring region of the A1 domain of von Willebrand Factor (UniProt: P04275), a protein essential for the regulation of hemostasis. In this case, however, the peptide of 19 residues was selected not on the basis of the sliding window analysis, but because it corresponds to an amphipathic secondary structure element (mainly α-helicoidal) of the A1 domain with a hydrophilic/cationic face and a hydrophobic face (Figure S2).

### 2.2. Antimicrobial Activity

**Figure 2:**
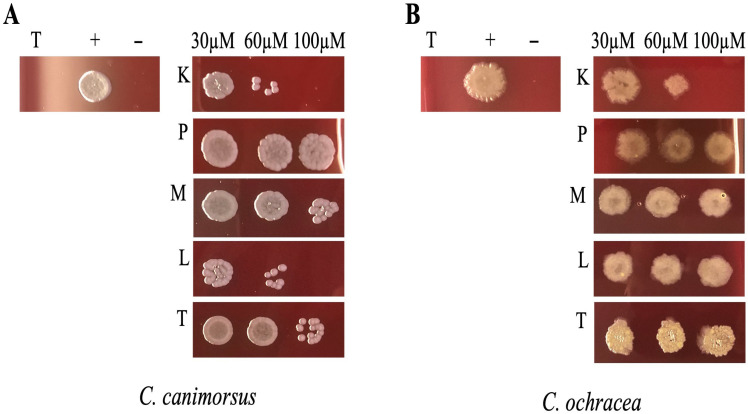
Effect of the different antimicrobial peptides on *C. canimorsius* and *C. ochracea* growth. Panel (**A**) shows the spots of *C. canimorsus*, while panel (**B**) shows those of *C. ochracea*. The spots were performed after treatment of the two strains with the different peptides: KTL24 (K), PQR19 (P), MFP22 (M), LIR23 (L) and TPS032 (T) at 30, 60 and 100 µM. The (+) indicates the positive control (untreated cells), (−) indicates the negative control (cells treated with antibiotic erythromycin 10 µg/mL) and (T) stands for the growth medium.

To investigate the antimicrobial properties of the selected peptides against *C. canimorsus* and *C. ochracea*, we incubated bacterial cultures for 24 h in DMEM supplemented with 10% heat-inactivated human serum (DMEM + HIHS) with different concentrations of peptides and subsequently spotted them onto HIA 5% sheep blood (SB) agar plates. This method was chosen because the liquid growth medium DMEM + HIHS interfered with absorbance readings at 600 nm, thus affecting OD measurements. As illustrated in Figure 2, the peptides KTL24 and LIR23 showed the most significant antimicrobial activity. Specifically, for *C. canimorsus* (Figure 2A), at 60 µM of KTL24 and LIR23, the spots exhibited few colony formations, indicating an antimicrobial potential. Indeed, at 100 µM, complete inhibition of bacterial growth was observed. Conversely, colony formation was still visible for 100 µM of TPS032, PQR19 and MFP22.

For *C. ochracea* (Figure 2B), KTL24 resulted as the most effective peptide, showing a trend similar to that observed with *C. canimorsus*. At 60 and 100 µM, KTL24 markedly reduced bacterial survival, proving to be more effective than LIR23. The other peptides tested, PQR19, MFP22, and TPS032, did not exhibit notable antimicrobial activity against either bacterial strain.

The minimum inhibitory concentration (MIC) of the selected peptides against *F. johnsoniae* and the two Gram (+) strains *S. oralis* and *S. mutans* was determined using the broth microdilution method. The experiments were repeated in triplicate, and by narrowing the effective concentration ranges, we determined the minimum inhibitory concentration (MIC) values, as shown in Table 1.

These results confirm that KTL24 is the most promising peptide, with MIC values of 60 µM for *C. canimorsus* and 80 µM for *C. ochracea*. LIR23 also displayed notable activity, with an MIC of 80 µM for *C. canimorsus*, though it was less effective against *C. ochracea*. Interestingly, the lowest MIC value (80 µM) was observed for MFP22 against *F. johnsoniae*. In contrast, neither *S. mutans* nor *S. oralis* exhibited sensitivity to any of the peptides tested. These findings highlight that the most promising peptides, KTL24, LIR23, and MFP22, show antimicrobial activity specifically against Gram (−) Bacteroidota strains.

**Figure 3:**
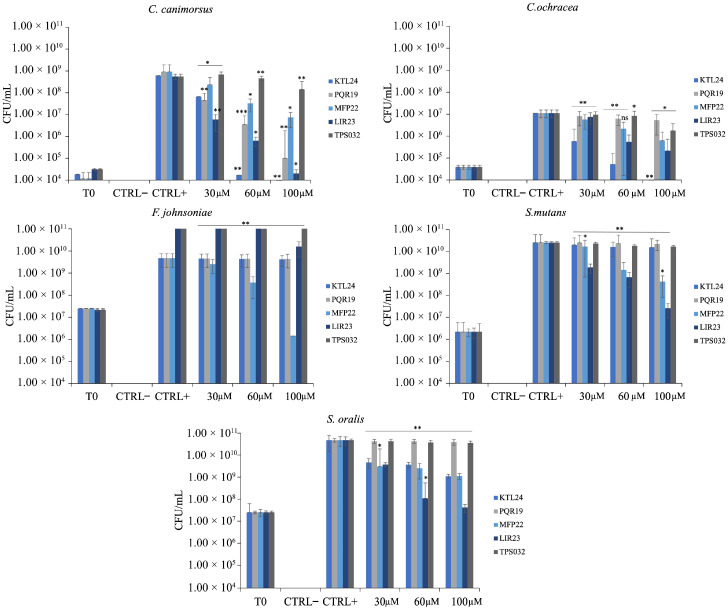
CFU/mL of the selected strains after treatment with the different peptides (0–100 µM). The T0 is the number of CFU/mL at time zero, while the negative control, after treatment with the erythromycin. The results are the average of three independent experiments. Statistical analysis was performed using a two-tailed *t*-test (ns: not significant, * *p* ≤ 0.05, ** *p* ≤ 0.01, *** *p* ≤ 0.001) versus CTRL+.

To complete the analysis of the antimicrobial properties and provide a more comprehensive understanding of the peptide’s potential and determine whether the MIC values indicated bacteriostatic or bactericidal activity, the CFU/mL values were calculated to evaluate the minimum bactericidal concentration (MBC). For each bacterial strain, prior to determining the MIC value, serial dilutions of the initial bacterial concentration at the beginning of the experiment (T0) were plated. After incubating bacteria without and with the peptides at concentrations of 30, 60, and 100 µM for 24 h and determining the MIC, 5 µL from each well was reinoculated into fresh medium and incubated overnight. The following day, serial dilutions of each sample were plated, and the CFU were counted. The results, Figure 3, show that for *C. canimorsus*, the most promising peptides were KTL24 and LIR23. KTL24 reduced CFU levels at approximately T0 counts at 60 µM, while LIR23 achieved this result at 100 µM. Interestingly, although the MIC of MFP22 exceeded 100 µM, a dose-dependent trend was observed, indicating antimicrobial potential for this peptide as well. For *C. ochracea*, the MIC results were confirmed, with KTL24 being the most effective peptide at 100 µM. Additionally, both LIR23 and MFP22 showed dose-dependent antimicrobial effects, further supporting their potential. For *F. johnsoniae*, only MFP22 exhibited antimicrobial activity, as CFU/mL values fell below those at T0 at 100 µM, confirming earlier results. In contrast, for *S. mutans* and *S. oralis*, no MBC values were observed. However, a dose-dependent trend for LIR23 and MFP22 was evident. In particular, at 100 µM, LIR23 reduced CFU/mL counts of *S. oralis* to levels nearly comparable to T0, suggesting that this peptide might have MIC/MBC values close to 100 µM against this strain.

These results, summarized in Table 2, highlight the MBC of specific peptides, especially KTL24, LIR23, and MFP22, against major sensitive bacterial strains, except for Gram (+) bacteria, which all showed MBC values higher than 100 µM.

### 2.3. Antibiofilm Properties

*S. mutans* and *S. oralis* are widely recognized as key bacterial species involved in biofilm formation within the oral cavity. *S. mutans* is well-known for its strong cariogenic potential, as it metabolizes dietary sugars into lactic acid, leading to localized acidification of the tooth surface below pH 5.5, which demineralizes enamel and promotes dental caries formation [22,23]. Similarly, *S. oralis*, although less directly associated with cariogenicity, plays a crucial role as an early colonizer in biofilm development by producing adhesion molecules and interacting with salivary glycoproteins, facilitating the attachment and stabilization of biofilms and promoting colonization by other species, including *S. mutans* [24].

**Figure 4:**
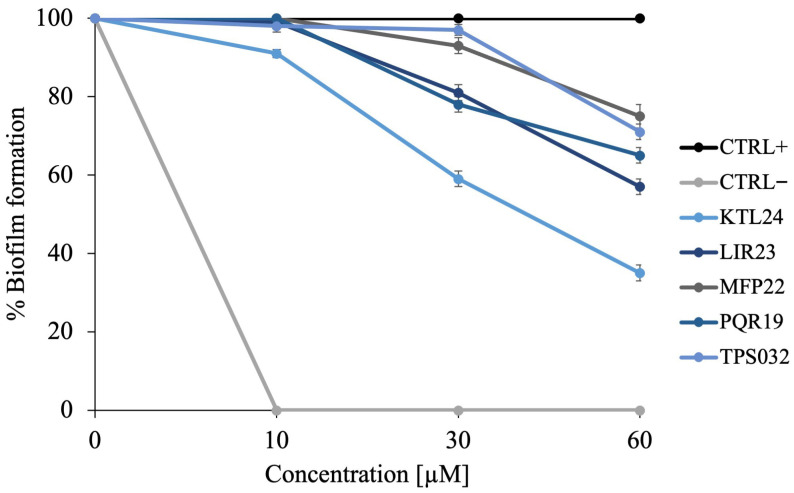
Colorimetric assay to evaluate the % of multispecies biofilm formation of *S. mutans* and *S. oralis*, at different concentrations of all the selected peptides (10, 30, 60 µM). The negative control is represented by Ciprofloxacin, and the positive control by untreated cells. The results are the average of three independent experiments.

Given their pivotal role in biofilm formation and pathogenesis, targeting *S. mutans* and *S. oralis* is crucial for the prevention and management of biofilm-associated oral diseases. To explore the antibiofilm potential of the tested peptides, multispecies biofilms were constructed through the co-incubation of these two bacterial strains. As shown in Figure 4, KTL24 exhibited promising antibiofilm activity, resulting in a 60% inhibition of biofilm formation at 60 µM. In contrast, the other peptides tested inhibited biofilm formation by 20–40%.

These findings suggest that KTL24 holds significant potential for disrupting biofilm development, particularly in multispecies contexts.

**Figure 5:**
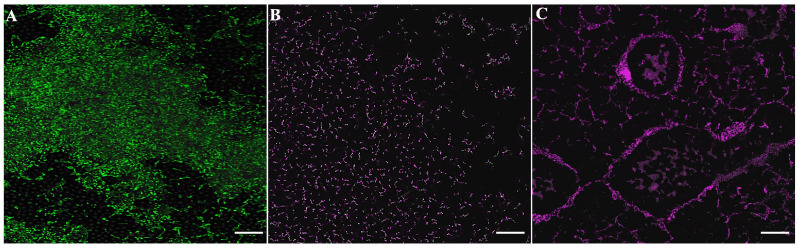
Confocal images of multispecies biofilms of *S. mutans* and *S. oralis*. Panel (**A**) shows the untreated biofilm, panel (**B**) the biofilm treated with ciprofloxacin, and panel (**C**) with KTL24. All panels represent confocal fluorescence microscopy. Scale bar: 10 μm.

Confocal microscopy was used to evaluate the effects of the treatments on the multispecies biofilm composed of *S. mutans* and *S. oralis*. In Figure 5A (untreated biofilm), green fluorescence (Syto9) predominates, indicating high cell viability and a dense biofilm structure. Treatment with ciprofloxacin (Figure 5B) results in reduced viability, as shown by the increase in red fluorescence (PI), although part of the biofilm structure remains intact. In contrast, the peptide KTL24 (Figure 5C) induces extensive cell death and a marked disruption of the biofilm architecture, evidenced by the strong prevalence of red fluorescence. These observations demonstrate a greater antibiofilm efficacy of KTL24 compared to the antibiotic.

### 2.4. Study of the Peptides’ Mechanism of Action

Since most AMPs exert their action mainly by damaging bacterial membranes and causing lysis through permeabilization, we tested whether this was also the case for our peptides. Therefore, detecting the permeability of the outer and inner membranes is a direct and important means to evaluate the efficacy of antibacterial molecules. Specifically, we tested the most interesting peptides, namely KTL24, LIR23 and MFP22, using fluorescent probes N-phenyl-1-naphthylamine (NPN) and propidium iodide (PI). NPN is a hydrophobic dye that dissolves sparingly in water with very low fluorescent emission. However, the fluorescence intensity increases sharply when NPN binds to nonpolar substances. The intact OM of Gram (−) effectively blocks NPN from bacteria to ensure that NPN cannot bind to the hydrophobic tail of phospholipids. In contrast, strong fluorescence emission can be detected when OM disruption occurs. Therefore, the change in fluorescence intensity of NPN may reflect the effectiveness of antibacterial molecules in increasing OM permeability [25].

**Figure 6:**
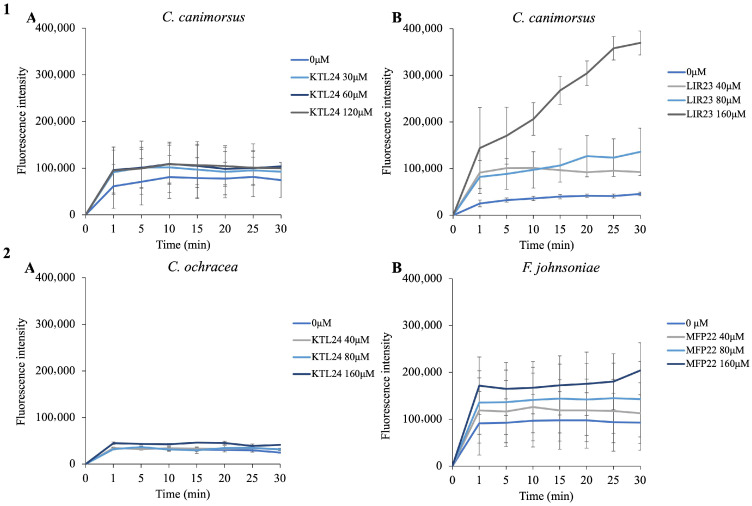
Fluorimetric assay to evaluate the integrity of the outer membrane (OM) of selected strains using the NPN probe. Bacterial cells were tested with concentrations equal to half the MIC, MIC, and double the MIC of the active peptides. Panel (**1A**) shows the results of *C. canimorsus* treated with KTL24; panel (**1B**) shows the results of *C. canimorsus* treated with LIR23; panel (**2A**) shows the results of *C. ochracea* treated with KTL24; panel (**2B**) shows the results of *F. johnsoniae* treated with MFP22. The results are the average of three independent experiments.

As shown in Figure 6(1A), KTL24 did not induce fluorescence emission in *C. canimorsus*. This is in contrast with the results shown in Figure 6(1B), where LIR23 treatment led to significant fluorescence emission. These findings suggest that LIR23 likely acts at the membrane level, disrupting the integrity of the OM. Conversely, KTL24 does not appear to affect the OM, as the probe fails to bind to phospholipids and does not emit fluorescence, even at concentrations twice the MIC. This observation is further supported by the data shown in Figure 6(2A), which shows an absence of fluorescence in *C. ochracea* following treatment with KTL24. In contrast, Figure 6(2B) highlights the action of MFP22 on the OM of *F. johnsoniae*, where it causes a disruption of membrane integrity. Against *C. canimorsus*, LIR23 appears to disrupt membrane integrity within one minute of incubation, while MFP22 exerts its effects on *F. johnsoniae* after 30 min. On the other hand, KTL24 does not seem to act at the membrane level, suggesting that its cellular target likely involves mechanisms other than membrane disruption.

For a more in-depth study of the cellular target also against Gram (+) bacteria, to determine the change in IM permeability after treatment with antibacterial molecules, we used a propidium iodine (PI) staining assay. PI is a red fluorescent nucleic acid stain that can bind to DNA and RNA between bases. Binding to DNA and RNA results in a 20- to 30-fold increase in PI fluorescence compared to aqueous solutions. Since PI is a membrane-impermeable stain, it can only label bacteria with a compromised inner membrane (IM).

**Figure 7:**
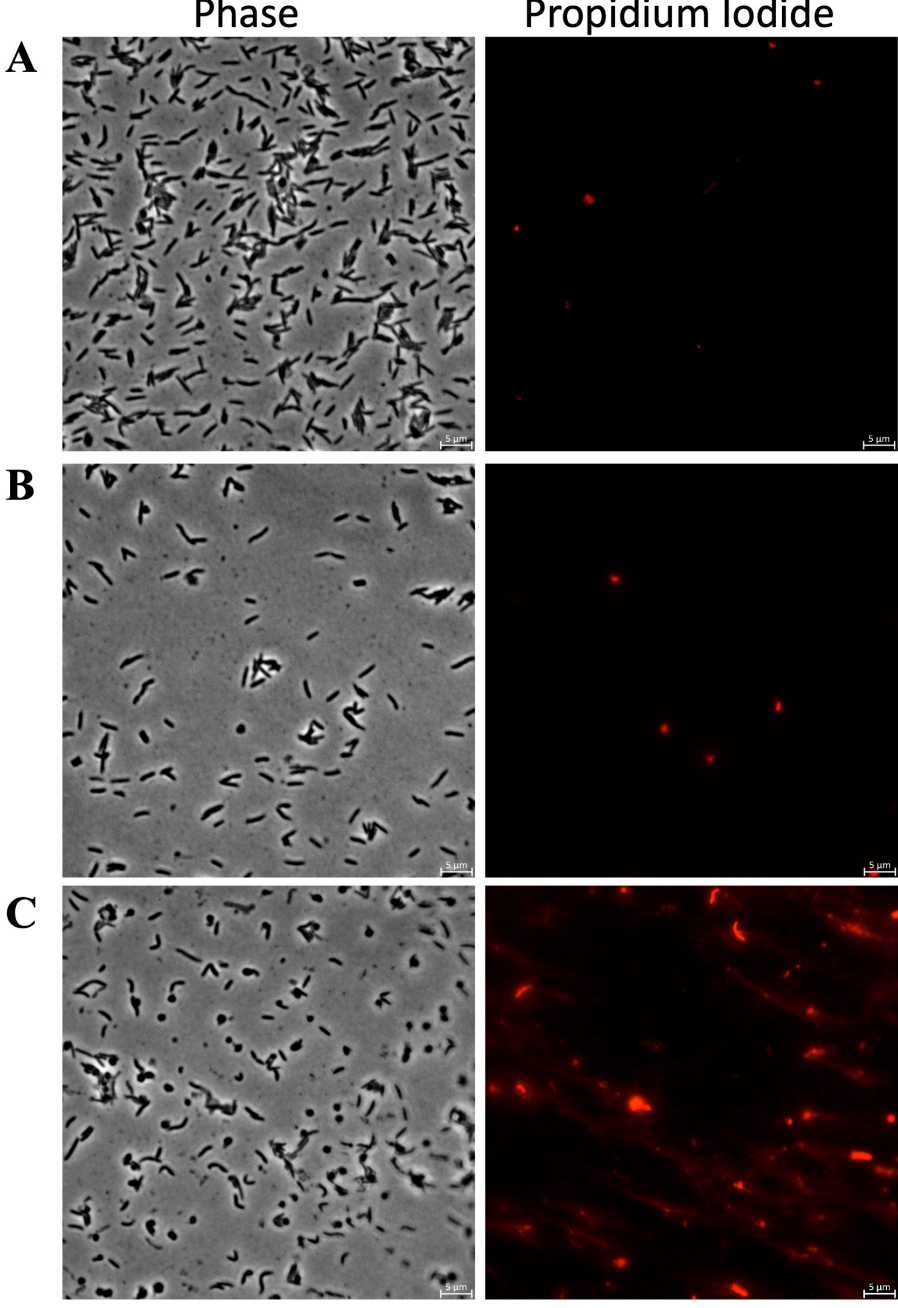
Evaluation of the antimicrobial mechanism of action of the most active peptides against *C. canimorsus* by PI staining and fluorescence microscopy. Panel (**A**) shows untreated cells, (**B**) cells treated with KTL24, (**C**) with LIR23 at sub-MICs. Scale bar: 5 µm.

Figure 7 shows fluorescence microscopy images of *C. canimorsus* treated with KTL24 and LIR23 at sub-MICs and stained with PI. Figure 7B shows that KTL24 does not seem to target the IM, as the red fluorescence from PI is comparable to that of the untreated control cells in Figure 7A. In contrast, Figure 7C shows a soft increase in red fluorescence in some cells after LIR23 treatment, suggesting an alteration of IM integrity. Interestingly, along with the membrane disruption, a clear change in cell morphology is observed, with the formation of spherical cells.

**Figure 8:**
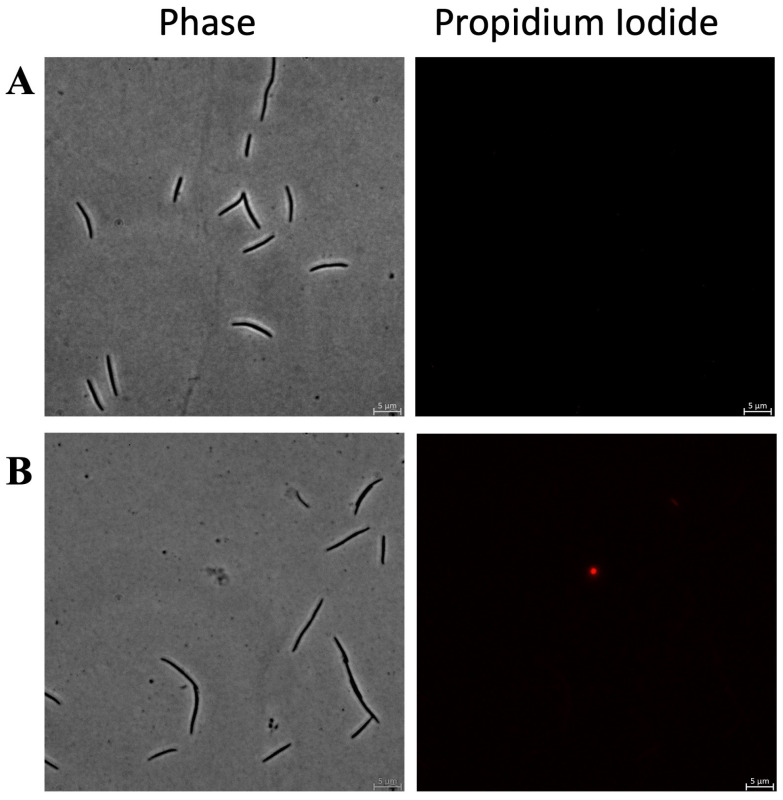
Evaluation of the antimicrobial mechanism of action of the most active peptide against *C. ochracea* by PI staining and fluorescence microscopy. Panel (**A**) shows untreated cells, (**B**) shows cells treated with KTL24 at sub-MICs. Scale bar: 5 µm.

Figure 8 shows the results of PI staining of *C. ochracea* treated with KTL24 at sub-MICs. As shown in Figure 8B, no fluorescence nor morphology change can be observed compared to the control in Figure 8A, confirming that KTL24 does not interact with the inner membrane in this strain.

**Figure 9:**
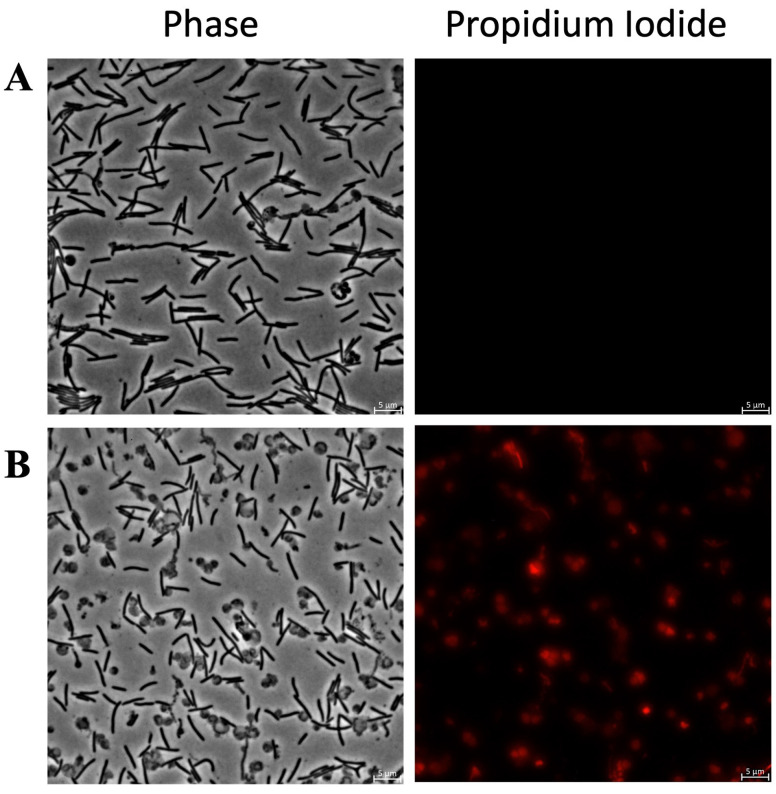
Evaluation of the antimicrobial mechanism of action of the most active peptide against *F. johnsoniae* by PI staining and fluorescence microscopy. Panel (**A**) shows untreated cells, panel (**B**) shows cells treated with MFP22 at sub-MICs. Scale bar: 5 µm.

Figure 9 highlights the results for *F. johnsoniae* treated with MFP22 at sub-MICs. In Figure 9B, treated cells show strong red fluorescence compared to the control (Figure 9A), along with pronounced morphological changes, including the formation of spherical cells. These alterations are more pronounced than those observed in *C. canimorsus* after treatment with LIR23 (Figure 7C).

Overall, these results confirm that LIR23 and MFP22 act on different Bacteroidota by disrupting membrane integrity, whereas KTL24 does not. Furthermore, the data reveal a striking phenomenon of bacterial morphological changes following treatment with LIR23 and MFP22, highlighting their impact on both membrane structure and cell shape. Since the peptides exhibited MIC values above 100 µM against *S. mutans* and *S. oralis*, but the CFU/mL analysis revealed a dose-dependent trend for the most promising peptides, namely KTL24, LIR23, and MFP22, we decided to assess potential damage to the membrane using these peptides at a concentration of 100 µM.

**Figure 10:**
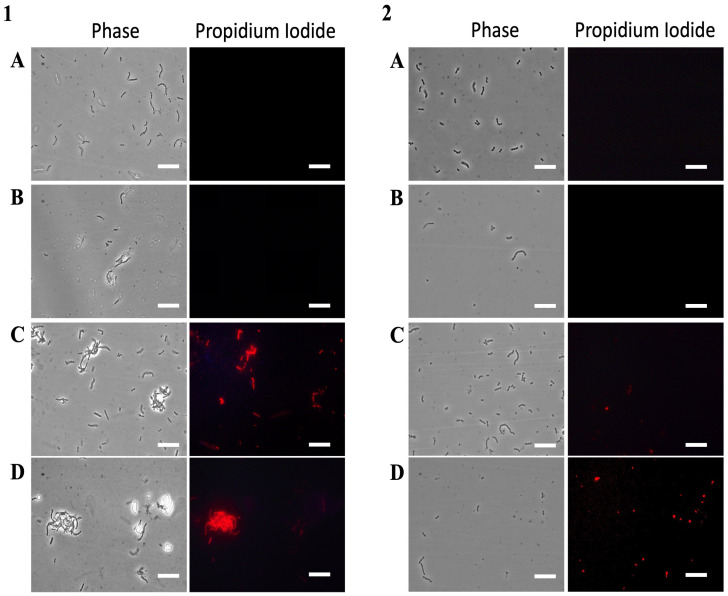
Evaluation of the antimicrobial mechanism of action of the most active peptides against *S. mutans* (**1**) and *S. oralis* (**2**) by PI staining and fluorescence microscopy. Panel (**A**) shows untreated cells, (**B**) cells treated with KTL24, (**C**) with LIR23 and (**D**) with MFP22 at sub-MICs. Scale bar: 5 µm.

As shown in Figure 10(1), treatment of *S. mutans* with KTL24 did not result in red fluorescence from PI staining (Figure 10B), confirming the earlier findings. In contrast, treatment with LIR23 (Figure 10C) and MFP22 (Figure 10D) shows the presence of red-fluorescent cells, indicating disruption of the membrane.

A similar trend is observed with *S. oralis* treated with these peptides at the same concentration (Figure 10(2)). However, unlike *S. mutans*, and consistent with the CFU/mL data, treatment of *S. oralis* with MFP22 did not result in intense red fluorescence. This finding confirms the lower efficacy of MFP22 against *S. oralis* compared to *S. mutans*.

### 2.5. Flavobacterium Growth with KTL24

**Figure 11:**
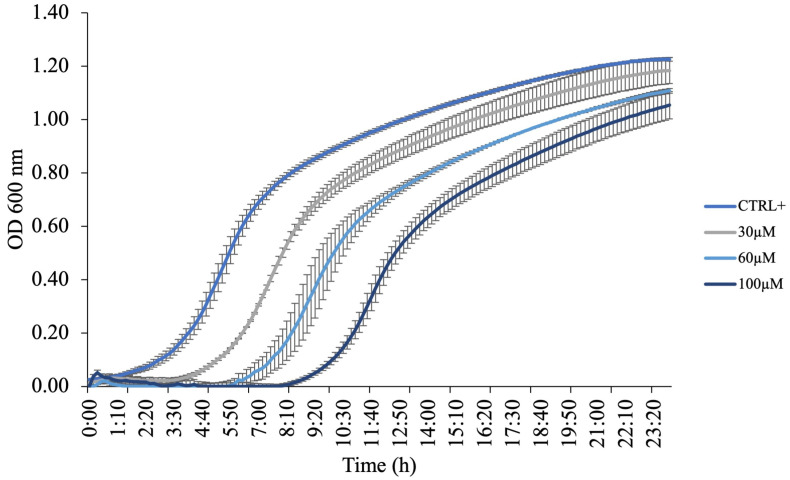
Evaluation of the antimicrobial mechanism of action of KTL24 against *F. johnsoniae*. The curves show how bacterial growth varies with or without KTL24 at different concentrations (0–100 µM). CTRL+ is the positive control, with untreated cells. Data are presented as the mean of three independent experiments.

Since KTL24 did not appear to interfere with bacterial membrane integrity, as shown by the absence of PI staining, we next evaluated its potential impact on bacterial growth dynamics through growth curve analysis. For this purpose, the Gram (−) environmental strain *F. johnsoniae* UW101 was selected as a model organism. *F. johnsoniae* was incubated at 30 °C for 24 h in the presence or absence of KTL24 at concentrations ranging from 0 to 100 µM. As shown in Figure 11, increasing concentrations of KTL24 progressively delayed the onset of the exponential growth phase without significantly affecting overall bacterial proliferation. This growth delay is consistent with previous findings indicating a lack of membrane permeabilization.

The observed growth modulation suggests that KTL24 exerts its antimicrobial activity through mechanisms distinct from membrane disruption. One plausible hypothesis is that the peptide interferes with intracellular processes critical for bacterial replication, such as DNA synthesis or protein translation, although the comparable slopes observed during the exponential phase suggest that these processes are not strongly inhibited once active growth is initiated. Alternatively, KTL24 may alter membrane selectivity, affecting the uptake of key nutrients or ions, thus primarily extending the lag phase and transiently impairing bacterial growth kinetics. Moreover, the activation of bacterial stress response pathways cannot be ruled out, potentially contributing to the delayed onset of exponential growth without causing bactericidal effects. These findings support the notion that the antimicrobial mechanism of KTL24 does not rely on membrane lysis but rather on early adaptive or regulatory processes and subtler intracellular or regulatory targets, warranting further mechanistic investigations.

### 2.6. Peptides Antioxidant Activity

**Figure 12:**
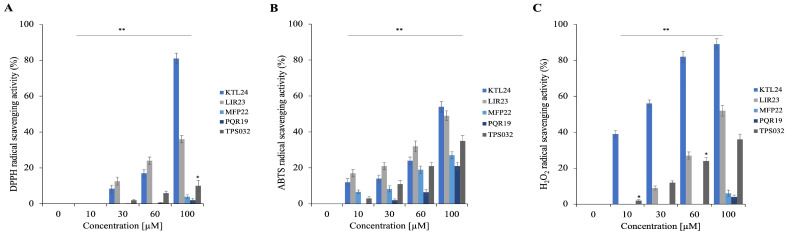
Determination of the antioxidant activity of selected peptides. Panel (**A**) shows the DPPH, (**B**) ABTS radical scavenging activity obtained after 20 and 10 min of incubation, and reported as % of DPPH and ABTS removed compared to the control. Panel (**C**) shows the hydrogen peroxide scavenging activity, measured after 30 min of incubation and reported as % of H_2_O_2_ removed compared to the control. Data were presented as the mean of three independent experiments. Statistical analysis was performed using a two-tailed *t*-test (* *p* ≤ 0.05, ** *p* ≤ 0.01) versus 0 µM samples.

To further explore the properties of the selected peptides, we evaluated their antioxidant potential using three different in vitro assays. These assays measure the peptides’ scavenging capabilities against DPPH, ABTS, and hydrogen peroxide (H_2_O_2_) radicals. The results presented in Figure 12A illustrate the scavenging activity against the DPPH radical, in Figure 12B are shown the results for ABTS, and in Figure 12C those regarding the activity against H_2_O_2_. Notably, KTL24 demonstrates the strongest antioxidant properties, effectively scavenging both DPPH and H_2_O_2_ radicals at percentages exceeding 80% at a concentration of 100 µM (Figure 12A,C).

### 2.7. Peptides Activity on Eukaryotic Cells

**Figure 13:**
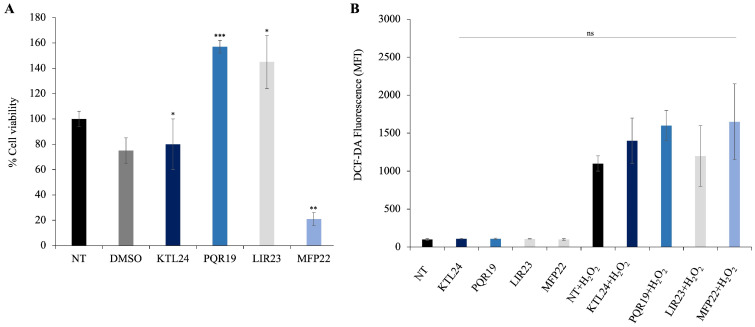
Effect of KTL24, PQR19, LIR23 and MFP22 on HaCat cells. (**A**) Viability of the cells was assessed by the MTT assay. The untreated cells (NT) were assumed as 100%. Percentage of cell survival in each condition was calculated by comparing with the untreated cells. (**B**) The effect of peptides on oxidative stress in HaCat cells was assessed by intracellular ROS reactive oxygen species (ROS) detection. The untreated cells (NT) were assumed as 100%. Percentage of fluorescence in each condition was calculated by comparing with the untreated cells. The assays were performed in three independent experiments. Statistical analysis was performed using a two-tailed *t*-test (ns not significant; * *p* ≤ 0.05; ** *p* ≤ 0.01; *** *p* ≤ 0.001) versus DMSO-treated cells.

To assess the impact of the selected peptides (KTL24, LIR23, PQR19, and MFP22) on the viability of eukaryotic epithelial cells, we performed MTT assays on HaCaT cells treated with each peptide at a concentration of 100 µM. The results (Figure 13A) indicate that the peptides PQR19 and LIR23 significantly increased cell viability. In contrast, the peptide MFP22 significantly decreased cell viability, demonstrating cytotoxic effects at higher concentrations. In addition, the peptide KTL24 did not affect cell viability compared to controls. These findings suggest that KTL24, PQR19, and LIR23 are promising candidates for potential medical applications, as they do not exhibit cytotoxicity and may even enhance cellular growth under certain conditions.

Regarding intracellular ROS levels, these were measured using DCF-DA fluorescence (Figure 13B). All tested peptides did not induce any significant increase in fluorescence compared to untreated controls, indicating that none of them triggered oxidative stress in HaCaT cells at the concentration of 100 μM. Notably, even MFP22, which reduced cell viability in the MTT assay, did not promote ROS production, suggesting that its cytotoxic effects are not mediated by oxidative stress. These findings further support the biocompatibility of the peptides and their potential suitability for biomedical applications.

However, when cells were pretreated with H_2_O_2_, a slight increase in fluorescence levels was observed, indicative of enhanced intracellular ROS production. Although modest, this effect confirms the sensitivity of the detection system and suggests that peptide exposure does not amplify the oxidative response induced by chemical stress. In other words, even in the presence of an external oxidative stimulus, the peptides do not worsen the cellular redox state, demonstrating a neutral or potentially protective behavior against H_2_O_2_-induced oxidative stress.

## 3. Discussion

In the broader context of the current antibiotic resistance crisis, this work addresses one of the major challenges faced by antimicrobial therapy: the urgent need for innovative strategies capable of overcoming resistance while preserving host biocompatibility.

Maintaining a healthy state is a fundamental priority in today’s world, and the effectiveness of pharmaceuticals plays a crucial role in achieving this goal. However, the overuse and misuse of antibiotics have led to an increasing threat of resistance among pathogenic microbes.

According to the 2019 Global Burden of Disease report, infections were responsible for approximately 14 million deaths, making them the second leading cause of mortality after ischemic heart disease. Bacterial pathogens alone accounted for 7.7 million deaths, with 1.3 million attributed specifically to antibiotic resistance, highlighting the urgent need for new antimicrobial strategies [26]. This underscores the pressing demand for alternative therapeutic approaches, especially for chronic and multifactorial oral infections such as periodontitis, caused by bacterial strains of the Bacteroidota phylum, which have recently also been associated with neurodegenerative diseases like Alzheimer’s [10].

Several antibiotic adjuvants, including β-lactamase inhibitors, efflux pump inhibitors, and outer membrane permeabilizers, show promise in overcoming antibiotic resistance [27]. In this context, antimicrobial peptides (AMPs) offer a promising avenue, not only as alternatives to conventional antibiotics but also as potentiators that can restore the efficacy of previously resistant antibiotics through synergistic mechanisms.

In this study, we characterized the antimicrobial activity of five cryptic peptides, four of human origin (KTL24, LIR23, PQR19, MFP22) and one of non-human origin (TPS032), against Gram (−) bacteria belonging to the Bacteroidota phylum, as well as Gram (+) strains commonly found in the oral microbiota, Firmicutes phylum. Specifically, the four human-derived peptides were selected from a pool of several dozen cryptic AMPs previously identified through an in silico analysis of the entire human proteome [7].

Our results highlight significant antimicrobial activity of three human peptides (KTL24, LIR23, and MFP22) against *C. canimorsus*, *C. ochracea*, and *F. johnsoniae*, with MIC values ranging between 60 and 100 µM, relatively low concentrations [28].

In contrast, Gram (+) oral bacteria (*S. mutans* and *S. oralis*) were less susceptible, showing MICs higher than 100 µM. However, LIR23 displayed partial efficacy, suggesting a potentially broader spectrum of action.

Using fluorescence-based assays with NPN and PI, we investigated the mechanism of action of the most promising peptides. The combination of these two fluorescent probes provided a comprehensive view: NPN revealed early outer membrane destabilization, while PI indicated subsequent damage to the inner membrane. LIR23 and MFP22 primarily acted via membrane-disruptive mechanisms, as evidenced by a significant increase in permeability of both outer and inner membranes. This is consistent with the most common AMP mechanism involving membrane disruption and bacterial death [29,30,31].

Conversely, KTL24 did not induce membrane permeabilization, suggesting an alternative mechanism of action. This hypothesis is supported by the observed delay in the exponential growth phase of *F. johnsoniae* treated with KTL24, likely due to interference with key intracellular processes such as protein synthesis or DNA/RNA metabolism [32]. Alternatively, KTL24 may alter membrane selectivity, affecting nutrient or ion uptake and temporarily impairing bacterial growth kinetics. Moreover, activation of bacterial stress response pathways cannot be ruled out, potentially contributing to the delayed onset of exponential growth without inducing bactericidal effects [33].

Some peptides, especially proline-rich AMPs (PrAMPs), are known to penetrate bacterial cells and inhibit EF-Tu or ribosomal activity to exert intracellular antibacterial effects [34]. While KTL24 does not belong to this class, our findings suggest it may exert a similar intracellular mode of action, warranting further investigation.

At the morphological level, strains treated with LIR23 and MFP22 exhibited clear structural alterations, such as a shift from rod-shaped to spherical morphology, particularly in *C. canimorsus* and *F. johnsoniae*. This further supports a direct effect on bacterial cell structures. Such changes were not observed in Gram (+) strains, consistent with the lower antimicrobial activity observed. Nevertheless, in Gram (+) strains, LIR23 and MFP22 also induced inner membrane damage, while KTL24 caused no appreciable membrane disruption.

Beyond antimicrobial activity, we also investigated additional intrinsic properties of the selected peptides, including antibiofilm activity.

The importance of oral biofilms in the pathogenesis of dental diseases is well established. Biofilms are complex, highly resistant microbial communities that form on tooth and mucosal surfaces, contributing to the development of cavities, plaque, and periodontal disease [35,36]. These microbial aggregates protect bacteria from immune responses and antibiotics, making them difficult to eradicate with conventional treatments [37,38].

Therefore, effective antibiofilm agents are essential for improved management of oral diseases, as they reduce bacterial adhesion, interfere with biofilm maturation, and limit bacterial virulence.

In this study, we developed a dual-species biofilm model (*S. mutans* + *S. oralis*) in the presence or absence of each peptide. Among them, KTL24 was the most effective, exhibiting approximately 60% biofilm inhibition. KTL24 may interfere with bacterial adhesion or early stages of biofilm formation. Additionally, confocal microscopy revealed its role in disrupting preformed biofilms. Using a live/dead staining kit, KTL24 induced strong red fluorescence in the interior of established biofilms, indicating bacterial killing. This ability to penetrate the biofilm and kill bacteria without dismantling the overall structure is especially interesting, as it prevents the systemic spread of bacteria released from disrupted biofilms [39]. These properties make KTL24 a particularly promising candidate for topical applications in the oral cavity.

Moreover, KTL24 exhibited strong antioxidant activity, neutralizing over 80% of DPPH and H_2_O_2_ radicals at 100 µM. Given the role of oxidative stress in chronic inflammation and the pathogenesis of periodontal diseases [40], this dual antimicrobial-antioxidant functionality further enhances KTL24’s relevance for oral health applications.

Finally, biocompatibility assays confirmed no significant cytotoxic effects of the tested peptides on HaCat epithelial cells, with only a slight reduction in cell viability observed for MFP22. These findings are encouraging for potential therapeutic development [41,42]. Moreover, the analysis of reactive oxygen species (ROS) production using the dichlorofluorescein diacetate (DCF-DA) assay showed that none of the tested peptides induced significant oxidative stress in HaCaT epithelial cells after 24 h of treatment at 100 μM. The measured fluorescence levels were comparable to those of the untreated and DMSO controls, indicating the absence of redox imbalance or oxidative damage. Interestingly, although MFP22 caused a reduction in cell viability in the MTT assay, this effect was not associated with an increase in ROS production, suggesting a possible cytotoxic mechanism that is not mediated by oxidative stress. In contrast, KTL24, LIR23, and PQR19 displayed good cellular tolerability, further confirming their biocompatibility profiles.

In summary, our results support the potential use of KTL24, LIR23, and MFP22 as candidates for treating bacterial infections, particularly those caused by Gram (−) pathogens of the Bacteroidota phylum. In the long term, these peptides could form the basis for preventive and therapeutic strategies against chronic oral diseases (e.g., periodontitis) and possibly contribute to the prevention of systemic disorders linked to the oral microbiome, such as Alzheimer’s disease, through targeted control of *P. gingivalis*.

Indeed, combining antimicrobial, antibiofilm, and antioxidant properties with low cytotoxicity, these peptides exemplify a multifunctional approach that aligns with emerging strategies aimed at reducing antibiotic misuse, limiting resistance development, and improving the management of chronic infections. Overall, our findings contribute to the growing body of evidence supporting antimicrobial peptides as valuable tools in the fight against antibiotic resistance, particularly for localized and biofilm-associated infections where traditional antibiotics often fail.

## 4. Materials and Methods

### 4.1. Antimicrobial Peptide (AMPs)

The peptides KTL24, 3095.88 g/mol (KTLRKWLKMFKKRQLELYLPKFSI); PQR19, 2363.86 g/mol (PQRMSRNFVRYVQGLKKKK); MFP22, 2770.42 g/mol (MFPRSFIKLLRSKVSRFLRPYK); LIR23, 2833.47 g/mol (LIRIPLHRVQPGRRILNLLRGWR) and TPS032, 1741.24 g/mol (RVLTHVFKCKLKLR) were all purchased from Caslo ApS (Kongens Lyngby, Denmark). All peptides were provided with a certificate of analysis and were characterized by mass spectrometry (MS) by the manufacturer. The experimentally determined molecular masses corresponded to the theoretical values. Peptide purity was ≥95%, as reported by the supplier.

### 4.2. Bacterial Strains

The Gram (−) bacterial strains used were *Capnocytophaga canimorsus strain Cc5, Capnocytophaga ochracea* human isolates and *Flavobacterium johnsoniae* UW101. The Gram (+) strains used were *Streptococcus oralis* CECT 8313 and *Streptococcus mutans* ATCC 35668.

### 4.3. Determination of Minimal Inhibitory Concentration

The minimum inhibitory concentration (MIC) of the selected peptides was determined using multiwell plate assays for *F. johnsoniae*, *S. mutans* and *S. oralis*, and spot assays for *C. canimorsus* and *C. ochracea*, following the Clinical and Laboratory Standards Institute guidelines (CLSI C 2008) [42]. For the MIC determination, bacterial cell suspensions were adjusted to 5 × 10^3^ cells per mL in 95 µL of the appropriate growth medium. Specifically, Dulbecco’s modified Eagle’s medium (DMEM; Invitrogen, Waltham, MA, US) supplemented with 10% (vol/vol) heat-inactivated human serum was used for *Capnocytophaga* strains, Casitone Yeast Extract (CYE) medium (10 g/L casitone, 5 g/L yeast extract, 8 mM MgSO_4_ and 10 mM Tris-HCl, pH 7.5) for *F. johnsoniae*, and TSB (AppliChem GmbH, Ottoweg, Darmstadt, Germany) for streptococcal strains. The peptide concentrations tested ranged from 0 to 100 µM.

After incubation, *Capnocytophaga* species were incubated for 24 h at 37 °C with 5% CO_2_, *F. johnsoniae* at 30 °C, and streptococci at 37 °C in a GasPak anaerobic system. Due to interference caused by DMEM+ heat-inactivated human serum at 600 nm, the wells containing *C. canimorsus* and *C. ochracea* cells were subjected to serial dilutions in PBS, and 3 µL from each dilution was spotted onto HIA plates containing 5% sheep’s blood. The spots were allowed to air dry before incubation under the same conditions as the liquid assay. The plates were then photographed against a black background to identify the MIC as the lowest peptide concentration that prevented visible growth [43]. For the other bacterial strains, the MIC was monitored by measuring absorbance at 600 nm. The MIC was determined as the lowest peptide concentration that resulted in no visible growth, based on the method described by Di Napoli et al. (2024) [44]. Erythromycin was used as a positive control for all bacterial strains.

### 4.4. Minimum Bactericidal Concentration (MBC) Assay

The MBC was determined by re-incubating 5 µL from each well of the MIC assay plate into 95 µL of fresh medium. After 24 h of incubation under the same conditions as the MIC assay (37 °C with 5% CO_2_ for *C. canimorsus* and *C. ochracea*, 30 °C for *F. johnsoniae* and 37 °C in a gas-pak system for *S.mutans* and *S. oralis*), the samples were subjected to serial dilutions in PBS. Aliquots of 10 µL from each dilution were plated on agar plates appropriate for the bacterial species: HIA with 5% blood for Capnocytophaga spp., CYE agar for *F. johnsoniae*, and TSA for streptococci. Plates were incubated under the same conditions for 24 h. The initial inoculum (5 × 10^3^ cells) from the MIC assay was plated as a “time-zero” control to confirm the starting bacterial concentration. The MBC was determined as the lowest peptide concentration that reduced viable bacterial growth by ≥99.9%, indicated by the absence of CFUs or a significant reduction in colony count compared to the initial inoculum.

### 4.5. Antibiofilm Assays

To evaluate the anti-biofilm properties of the selected peptides, a multispecies biofilm model comprising *S. mutans* and *S. oralis* was employed. The artificial saliva for biofilm formation was prepared by dissolving 1 g/L of ‘Lab-lemco’ powder, 2 g/L of yeast extract, 5 g/L of protease peptone, 2.5 g/L of porcine gastric mucin, 0.35 g/L of sodium chloride, 0.2 g/L of calcium chloride, and 0.2 g/L of potassium chloride in distilled water. After autoclaving, 1.25 mL of a 40% urea solution was added to the sterilized mixture. For biofilm formation, a 24-well plate was pretreated by adding 1 mL of artificial saliva to each well, followed by incubation at 37 °C for 4 h. After incubation, the saliva was aspirated, and the wells were gently washed with 10 mM PBS (pH 7.0) to remove any residual saliva. To initiate biofilm growth, TSB broth was supplemented with bacterial suspensions of *S. mutans* and *S. oralis* at a concentration of 10^6^ CFU/mL. Peptides at concentrations ranging from 10–60 μM were added to the wells. Biofilms were incubated anaerobically at 37 °C for 96 h.

For controls, wells containing only bacterial cells and growth medium were used as negative controls, while bacterial cells treated with ciprofloxacin served as positive controls. To evaluate the anti-biofilm activity, biofilms were stained with crystal violet dye after incubation. The OD of the stained biofilm was measured at 570 nm using a Multiskan microplate reader (Thermo Electron Corporation, Waltham, MA, USA) following established protocols [45]. The percentage of biofilm formation was calculated by comparing the OD values of peptide-treated samples to those of untreated samples, after normalizing the ODs to those of the planktonic growths [46]. For the biofilm disruption assay, a circular glass coverslip (thickness 0.13–0.16 mm; Deltek, Pozzuoli, Italy) was placed at the bottom of each well prior to biofilm formation. After treatment with KTL24 (60 μM) and incubation, bacterial viability within the adhered biofilm was assessed using the LIVE/DEAD™ BacLight™ Bacterial Viability Kit (Thermo Fisher Scientific, Waltham, MA, USA), following the manufacturer’s instructions. Confocal laser scanning microscopy was performed using a Zeiss LSM 800, and images were acquired to visualize and evaluate the distribution of live and dead cells within the biofilm structure.

### 4.6. 1-N-Phenyl Naphthylamine (NPN) Assay

The outer membrane (OM) permeabilizing activity of the peptides KTL24, LIR23, and MFP22 was evaluated using the 1-N-phenylnaphthylamine (NPN) assay, with minor modifications based on the protocol by Jia et al. [47]. In brief, cells of *C. canimorsus*, *C. ochracea*, *F. johnsoniae*, *S. mutans* and *S. oralis* were collected after overnight incubation under their respective optimal growth conditions, washed, and resuspended in 5 mM N-(2-hydroxyethyl)piperazine-N’-ethanesulfonic acid (HEPES) buffer (pH 7.2) to an optical density at 600 nm (OD_600_) of 0.5 ± 0.02. For the assay, 50 µL of each peptide at various concentrations (half the MIC, MIC, and double the MIC) was added to 100 µL of the bacterial suspension in black 96-well fluorescence plates. To this mixture, 50 µL of NPN at a final concentration of 40 µM was added. Control wells contained only HEPES buffer, bacterial cells, and NPN without peptides. Fluorescence measurements were performed using a fluorimeter, with excitation and emission wavelengths set at 350 nm and 420 nm, respectively. Fluorescence intensity was used as an indicator of OM permeabilization.

### 4.7. Fluorescence Microscopy

To study the alteration of the inner membrane (IM), under optimal growth conditions and in the absence of light, bacterial cells at a concentration of 10^7^ CFU/mL were incubated for 4 h, either with or without the peptides KTL24, LIR23, and MFP22 at sub-MICs (40–100µM). Following incubation, 10 µL of each sample was treated with propidium iodide (PI) at a final concentration of 20 μg/mL. The samples were subsequently analyzed using fluorescence microscopy, following the protocol described by Castagliuolo et al., to assess membrane integrity [48].

### 4.8. Growth Curve of F. johnsoniae with KTL24

A culture of *F. johnsoniae* was started by inoculating a single colony into CYE broth and incubating at 30 °C with orbital shaking until the exponential phase was reached (OD600 ≈ 0.5). The culture was then diluted in fresh CYE to obtain an initial OD of 0.1 in each well of a 96-well plate.

Growths were started with or without the presence of KTL24 at concentrations ranging from 0 to 100 µM. Control wells contained untreated bacteria. The plate was incubated at 30 °C with continuous linear shaking (567 cpm), and growth was monitored by measuring the optical density at 600 nm (OD600) every 10 min for 24 h, using an EPOCH2 Microplate Reader (BioTek, Milano, Italy).

### 4.9. DPPH Radical Scavenging Assay

The DPPH (2,2-diphenylpicrylhydrazyl hydrate) radical scavenging activity was assessed as described by Kedare and Singh (2011) [49]. Peptide solutions at concentrations ranging from 0 to 100 μM were prepared in 100% methanol. Freshly prepared DPPH solution (0.1 mM) was added to the peptide solutions to a final volume of 1 mL, ensuring the initial absorbance of the DPPH solution was ≤1.0. The reaction mixtures were incubated at 25 °C for 30 min in the dark. Absorbance was measured at 517 nm using a spectrophotometer. The scavenging activity was calculated using the following equation: DPPH radical scavenging activity (%) = (1 − AS/AC) × 100, where AS is the absorbance of the reacted mixture of DPPH with the extract sample, and AC is the absorbance of the DPPH solution.

### 4.10. ABTS Radical Scavenging Assay

The ABTS radical cation scavenging activity was determined according to the method of Re et al. (1999) [50], with modifications. A stock ABTS solution was prepared by mixing 7 mM ABTS with 2.45 mM potassium persulfate and incubating the mixture in the dark at room temperature for 16 h. The stock solution was diluted with phosphate-buffered saline (PBS) to achieve an absorbance of 0.72 ± 0.02 at 734 nm.

Peptide solutions (0–100 μM) were added to 1 mL of the ABTS solution, and the mixtures were incubated in the dark at room temperature for 10 min. Absorbance at 734 nm was measured using a PharmaSpec UV/Vis spectrophotometer (Shimadzu, Duisburg, Germany). The scavenging activity was calculated as:

ABTS^+^ radical scavenging activity (%) = (1 − AS/AC) × 100, where AC is the absorbance of the ABTS solution, and AS is the absorbance of the sample at 734 nm.

### 4.11. Hydrogen Peroxide Scavenging Assay

The ability of peptides to scavenge hydrogen peroxide was evaluated following the method of Beers and Sizer (1952) [51] with modifications from Petruk et al. (2018) [52]. Fresh hydrogen peroxide solution (50 mM potassium phosphate buffer, pH 7.0, containing 0.036% H_2_O_2_) was prepared. Peptide solutions (0–100 μM) were added to 500 μL of the hydrogen peroxide solution and incubated at 20 °C for 30 min.

After incubation, the mixtures were centrifuged at 13,000× *g* for 1 min, and the supernatant was analyzed for remaining hydrogen peroxide by measuring absorbance at 240 nm. The scavenging activity was calculated using the equation: peroxide removed (%) = (1 − AS/AC) × 100, where AC is the absorbance of 1 mL of hydrogen peroxide solution, and AS is the absorbance of the sample at 240 nm.

### 4.12. Cytotoxicity Assay

HaCaT cells were cultured in Dulbecco’s Modified Eagle Medium (DMEM), supplemented with 10% fetal bovine serum, 2 mM of L-glutamine, and 1% penicillin–streptomycin. Cells were grown at 37 °C with 5% CO_2_. 2 × 10^4^ cells were seeded into a 96-well plate and cultured for 24 h. Then, the medium was replaced with 100 μL of fresh medium containing KTL24, PQR19, LIR23 or MFP22 at a final concentration of 100 μM/well. After 24 h of incubation at 37 °C, the resulting insoluble formazan salts were solubilized in 0.04 M HCl in anhydrous isopropanol and quantified by measuring the absorbance at λ = 570 nm (SynergyTM H4, Agilent BioTek, Santa Clara, CA, USA) [53].

### 4.13. Reactive Oxygen Species (ROS) Detection Assay

2 × 10^4^ of HaCaT cells were seeded in a 96-well plate. After 24 h, the culture media were changed with fresh DMEM without phenol red supplemented with 10% FBS and with KTL24, PQR19, LIR23 or MFP22 at a final concentration of 100 μM/well, with or without H_2_O_2_. After 24 h, the culture media were replaced with fresh DMEM without phenol red, supplemented with 2% FBS. The fluorescence intensity (485/535 nm) was measured using a Synergy H4 Hybrid Microplate reader (Agilent, Santa Clara, CA, USA) [53].

### 4.14. Statistical Analysis

All experiments were performed in triplicate, and data were expressed as the mean. For statistical analysis, data were analyzed using a two-tailed paired-samples *t*-test. Differences were considered statistically significant at *p* values ≤ 0.05 (ns not significant; * *p* ≤ 0.05, ** *p* ≤ 0.01, *** *p* ≤ 0.001, **** *p* ≤ 0.0001).

## 5. Conclusions

In conclusion, our study demonstrates that the human-derived peptides KTL24, LIR23, and MFP22 possess a range of beneficial biological activities, including antimicrobial effects against both Gram (−) and Gram (+) bacteria, antibiofilm capabilities, and antioxidant properties, while maintaining minimal cytotoxicity on eukaryotic cells.

KTL24, in particular, stands out for its strong antibiofilm and antioxidant activities, combined with its ability to interfere with bacterial growth without exerting harmful effects on host cells. The confocal microscopy analyses confirmed the ability of KTL24 to penetrate and act within structured biofilms, inducing bacterial killing without causing dispersal of biofilm biomass, a particularly advantageous feature for preventing systemic bacterial dissemination. Mechanistic investigations revealed that LIR23 and MFP22 primarily act through membrane disruption, whereas KTL24 likely exerts its effects by targeting intracellular pathways, potentially involving protein synthesis regulation. Additionally, ROS quantification in HaCaT cells demonstrated that none of the peptides induced oxidative stress, further supporting their biocompatibility and safe cellular profile. Together, these results suggest that cryptic peptides derived from the human proteome represent a promising reservoir for the development of novel antimicrobial and bioactive agents. Future studies focusing on in vivo efficacy, stability optimization, and formulation strategies will be essential to advance these peptides toward clinical application.

## Figures and Tables

**Table 1:** Peptides MIC against *C. canimorsus*, *C. ochracea* and *F. johnsoniae.* The results are the average of three independent experiments.

	MIC [µM]		
Peptides	*C. canimorsus*	*C. ochracea*	*F. johnsoniae*
**KTL24**	≥60	80	>100
**PQR19**	>100	>100	>100
**MFP22**	>100	>100	80
**LIR23**	80	>100	>100
**TPS032**	>100	>100	>100

**Table 2:** Representation of the results of the minimum bactericidal concentration (MBC) after treatment with all selected peptides. The results are the average of three independent experiments.

Peptides	MBC [µM]		
	*C. canimorsus*	*C. ochracea*	*F. johnsoniae*
**KTL24**	70	100	>100
**PQR19**	>100	>100	>100
**MFP22**	>100	>100	100
**LIR23**	100	>100	>100
**TPS032**	>100	>100	>100

## Data Availability

Data are contained within the article.

## Supplementary Materials

The following supporting information can be downloaded at https://www.mdpi.com/article/10.3390/antibiotics15010080/s1. Figure S1: Sliding window analysis of the human proteins; Figure S2: Crystallographic structure of the A1 domain of the human von Willebrand Factor.
