# Supplementary material for: Study of Human Antimicrobial Peptides Active Against Some Bacteroidota Species of the Oral Cavity
Source: Antibiotics (Basel). 2026 Jan 13;15(1):80. doi: 10.3390/antibiotics15010080 (PMC12838075; doi:10.3390/antibiotics15010080)
Supplement: Supplementary file 1 [file antibiotics-15-00080-s001.zip › antibiotics-4077868-supplementary.pdf]

A

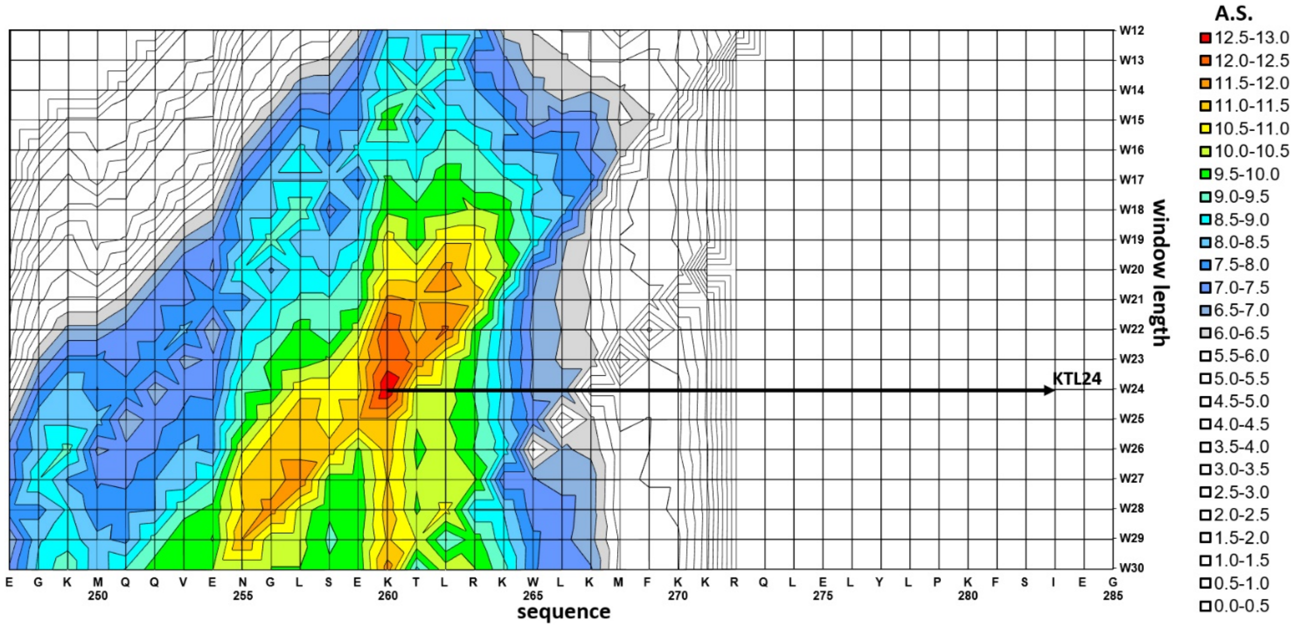
A.S.
12.5-13.0
12.0-12.5
11.5-12.0
11.0-11.5
10.5-11.0
10.0-10.5
9.5-10.0
9.0-9.5
8.5-9.0
8.0-8.5
7.5-8.0
7.0-7.5
6.5-7.0
6.0-6.5
5.5-6.0
5.0-5.5
4.5-5.0
4.0-4.5
3.5-4.0
3.0-3.5
2.5-3.0
2.0-2.5
1.5-2.0
1.0-1.5
0.5-1.0
0.0-0.5
KTL24
W12 W13 W14 W15 W16 W17 W18 W19 W20 W21 W22 W23 W24 W25 W26 W27 W28 W29 W30
window length
E G K M Q Q V E N G L S E K T L R K W L K M F K K R Q L E L Y L P K F S I E G
250 255 260 265 270 275 280 285
sequence

B

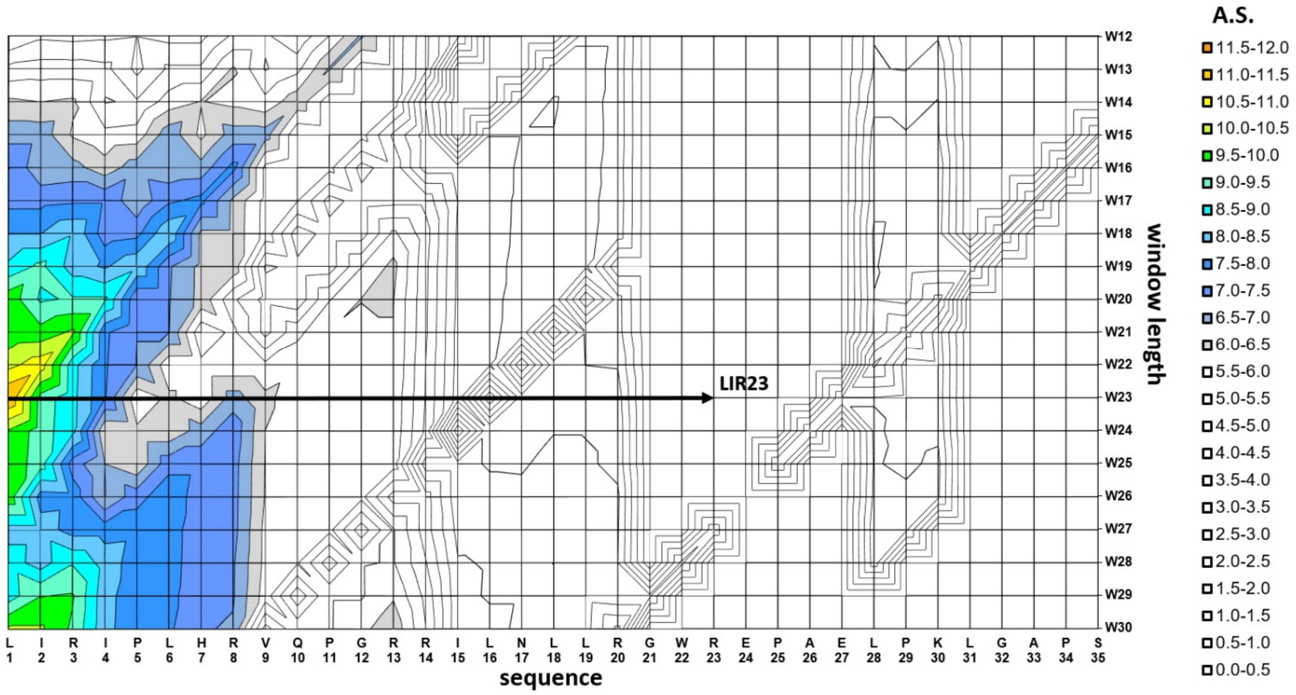
A.S.
11.5-12.0
11.0-11.5
10.5-11.0
10.0-10.5
9.5-10.0
9.0-9.5
8.5-9.0
8.0-8.5
7.5-8.0
7.0-7.5
6.5-7.0
6.0-6.5
5.5-6.0
5.0-5.5
4.5-5.0
4.0-4.5
3.5-4.0
3.0-3.5
2.5-3.0
2.0-2.5
1.5-2.0
1.0-1.5
0.5-1.0
0.0-0.5
LIR23
W12 W13 W14 W15 W16 W17 W18 W19 W20 W21 W22 W23 W24 W25 W26 W27 W28 W29 W30
window length
L I R I P L H R V Q P G R R I L N L L R G W R E P A E L P K L G A P S
1 2 3 4 5 6 7 8 9 10 11 12 13 14 15 16 17 18 19 20 21 22 23 24 25 26 27 28 29 30 31 32 33 34 35
sequence

C

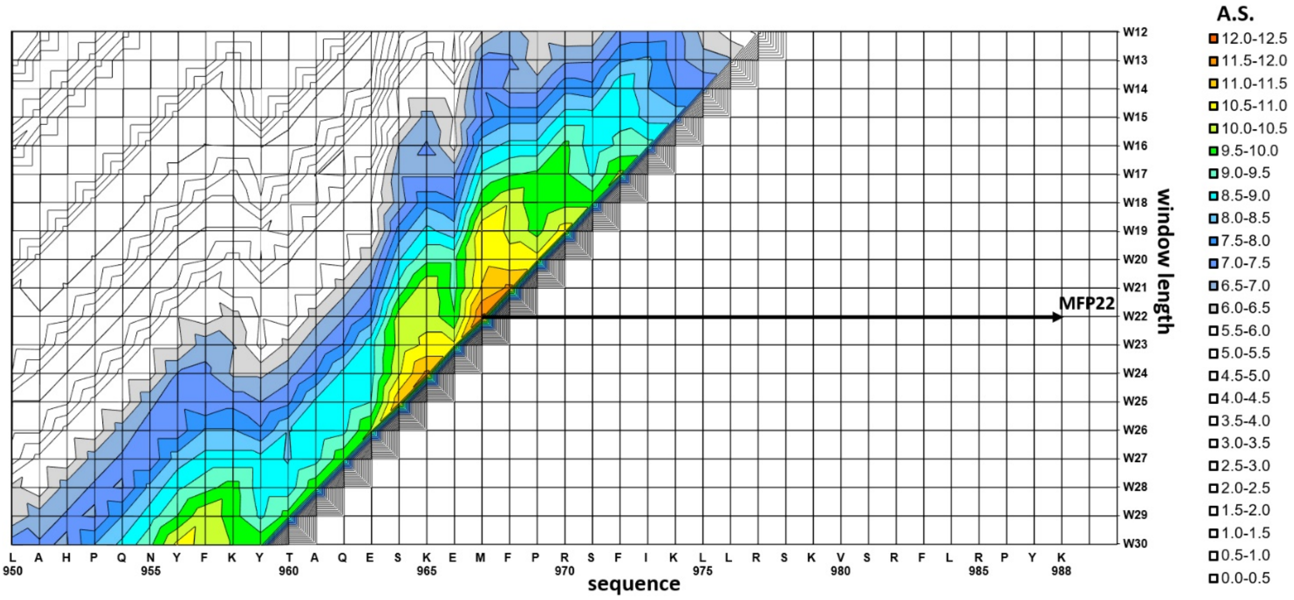

Figure. S1. Sliding window analysis of the human proteins (A) Plasma Serine Protease Inhibitor (UniProt: P05154; mature form), (B) Napsin A (UniProt: O96009; zymogen), and (C) SCUB1, Signal peptide, CUB and EGF-like domain-containing protein 1 (UniProt: Q8IWY4; precursor). Absolute Scores (A.S.) of the peptides are reported as function of the position (*x* axis) and window length (*y* axis). A.S. values were calculated using the hydrophobicity scale "Parker-Arg0" and the parameters derived for *S aureus* C623 described in Pane et al. 2017 (DOI: 10.1016/j.jtbi.2017.02.012).

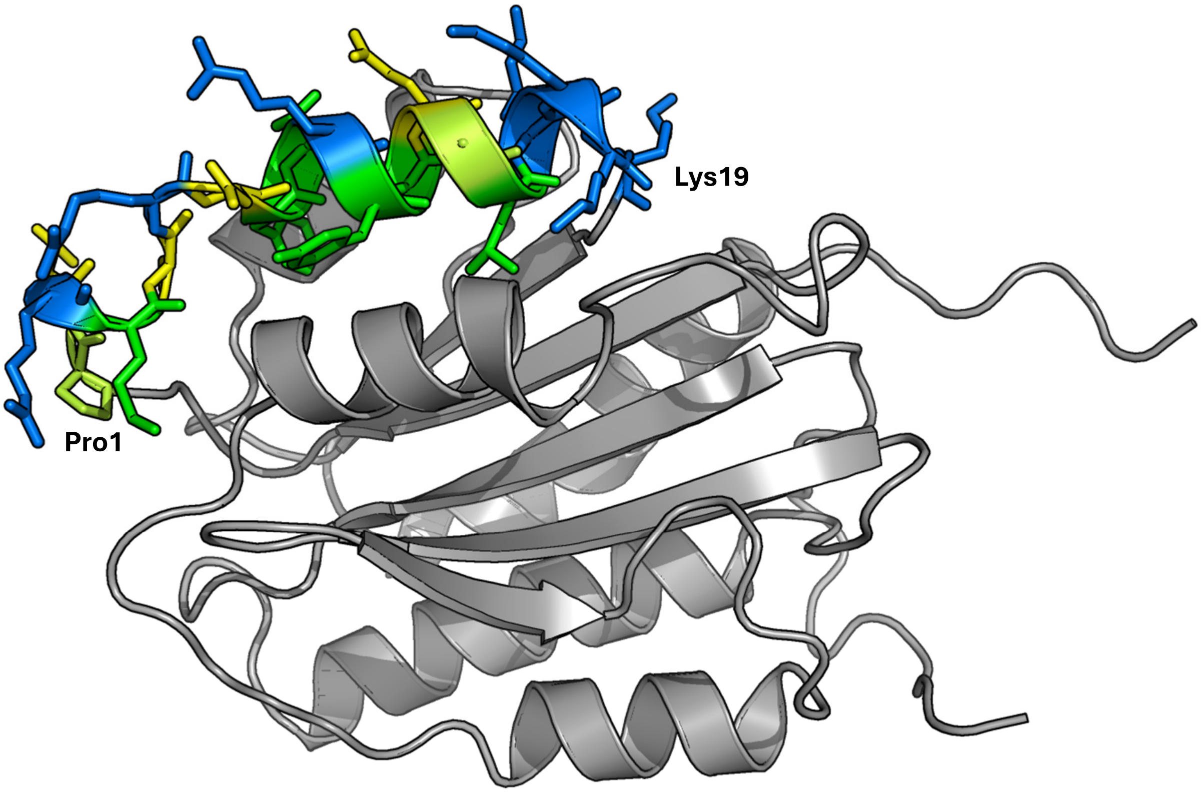

Figure. S2. Crystallographic structure of the A1 domain of the human von Willebrand Factor (PDB code: 1AUQ). The residues of PQR19 are shown as sticks and colored by residue type: blue, basic; yellow, polar uncharged; green, hydrophobic; greenish yellow, borderline.
